# Voices of Australian Mature-Age Bachelor of Counselling Students: Telling Stories of Learning and Teaching Transitions

**DOI:** 10.1007/s10447-023-09508-1

**Published:** 2023-04-21

**Authors:** Peyman Abkhezr, Debra Bath

**Affiliations:** 1 School of Applied Psychology, Griffith University, Gold Coast, Australia; 2 School of Applied Psychology, Griffith University, Brisbane, Australia

**Keywords:** Bachelor of counselling, Narrative inquiry, Student voice, Voice-centred relational analysis, Higher education transitions

## Abstract

The COVID-19 pandemic imposed transformations on the higher education context of the twenty-first century that adversely impact students’ learning in certain disciplines. On a mission to adopt ethics of care in research and practice, this research focuses on counselling education and its unique characteristics, by signifying counselling students’ voices in such changing contexts. A qualitative exploratory multiple case study design informed by narrative inquiry was used, followed by a voice-centred relational method of analysis. Findings revealed voices, relationships, dominant narratives and power relations that influence counselling students’ learning experiences. Implications for future research and practice for counselling education are highlighted.

## Introduction

Over the past few decades, forces such as globalisation, technological advances, and the evolving political and economic context of the twenty-first century have realigned some higher education priorities (Cannella & Koro-Ljungberg, 2017). This rapidly changing educational context was further impacted by the COVID-19 pandemic and the resulting changes that occurred within educational institutions (Aristovnik et al., 2020; Toquero, 2020). The Australian higher education sector was not an exception and lost more than 17,000 jobs and over $3.8 billion of revenue during 2020–2021, potentially losing more in revenue until 2023 (Universities Australia, 2021). These contextual and practical limitations followed by other ‘pandemic-related restrictions’, such as lockdowns, travel limitations and social distancing, resulted in dramatic pedagogical changes (Darby, 2020; Oyedotun, 2020). Many believe the pandemic has significantly legitimised a move to online teaching (Ewing, 2021). Adopting new virtual and hybrid ways of teaching and learning by higher education institutions raises concerns for some students, disciplines, and contexts (Li et al., 2021; Potra et al., 2021). While these changes raise serious considerations for student equity and inclusion (Organisation for Economic Co-operation & Development, 2020), they variously impact students’ experiences in different disciplines. Tertiary Education Quality and Standards Agency (TEQSA) of the Australian Government reported significant issues associated with online learning in particular disciplines (TEQSA, 2020), including practical disciplines that substantially rely on “a face-to-face mode of learning” (p. 12). One such discipline that traditionally relied on face-to-face delivery is counselling education.

### Counselling Education in Australia

In 2021, there were over 80 counselling training programs across Australia (in Diploma, Bachelor, Graduate Diploma and Master levels). Counselling education requires practical spaces that mirror the real-world practices of counselling and facilitate training in practical areas such as the interpersonal dimensions of client-counsellor relationship that have been historically highlighted as the major bedrock of counselling efficacy and outcome (Duff & Bedi, 2010; Wampold & Imel, 2015). In order to become competent upon graduation, counselling students need to sufficiently practice, demonstrate and reflect on their development of core skill-based aspects of their learning. Learning spaces that promote and facilitate reflectivity and reflexivity in counselling education have been highlighted as vital in the technology-led twenty-first century (Dixon & Chiang, 2019; Luft & Roughley, 2016). The kind of space that facilitates the teaching and learning of counselling as a reflective and practical discipline also emphasises consistent and continuous ‘counselling supervision’ as an integral part of counselling education (Hess et al., 2008). Counselling supervision is one of the most prominent and continuous aspects of counselling education and may be utilised in many ways across a variety of courses across the program. In other words, the educators delivering a counselling program often develop some form of a professional relationship and working alliance with their learners (e.g. while providing feedback) in which many dimensions of counselling supervision are mirrored. As such, the boundaries of counselling education and supervision overlap at times (Moir-Bussy, 2008). Such supervisory-educational relationships aim to nourish counselling students’ ongoing learning by facilitating reflective spaces that help them thrive and inform their evolving professional counselling identities as they transition through their learning journeys (Moir-Bussy, 2008).

Counselling practice, education and training encountered several unique challenges and complications during the pandemic (Tudor et al., 2021). Both educators and students felt significantly affected by such challenges. Considering the contextual, practical and historical backgrounds of the counselling discipline, which face transformations as a result of the pandemic and ongoing advances in technology, it becomes important to re-visit the context of counselling education from the perspective of students as a major stakeholder whose voices are often neglected or marginalised (Alvarado et al., 2019; Greene & Flasch, 2019). Given the bulk of counselling research focuses on counselling effectiveness, outcomes and clients’ experiences, counselling education research remains limited. The latest annual review of counsellor education and supervision reviewed 139 research articles in this area and outlined that despite a notable increase in research that focuses on counselling education, and online teaching and supervision, a focus on counselling students’ experiences of learning and supervision requires further attention (La Guardia, 2021). Counsellor educators and researchers need to reflect on the possibilities and limitations that surround the changing context of counselling education and remain sensitive to future generation of counsellors’ needs and priorities. The first step in this path could involve attending to diverse learning and teaching experiences of counselling students, and their transitions into and out of counselling programs, sometimes reflected through the ‘journey’ metaphor.

### Journey Metaphors

Journey metaphors are not uncommon in counselling and psychotherapy practice (Tay, 2011; White, 2002). Although such metaphors are often used to characterise a client’s process of counselling experience, journey metaphors might also be helpful to be considered for counselling students’ transitional learning experiences and educational journeys. Journey metaphors also emerge in career development literature (Inkson, 2015). As people make sense of their careers, their narratives reflect the discursive influences that are informed by the notion of a ‘journey’ (Tay, 2011). People rely on such metaphors to story their experience through imagery that encompasses elements of “mobility, immobility, direction, adaptation, and unpredictability” (Inkson, 2015, p. 35). As counselling students’ career and professional identities emerge and evolve throughout the different phases of their studies, journey metaphors reflect their learning experiences. A major component of reflective teaching practices in counselling education (Taylor, 2020) is to reflect on students’ learning experiences by attending to their voices.

### Student Voice and Reflective Teaching Practices in Counselling Education

Student’s voice reflects the possibility for students to story an opinion, raise concerns, provide feedback, problematise, participate, authorise and transform educational practices and systems to improve learning and teaching outcomes (Bennett & Kane, 2014; Flint et al., 2017; Young & Jerome, 2020). Such activities may range from small classroom initiatives that aim to incorporate student feedback (Fielding, 2004) to larger-scale transformations within the higher education sector (Flint et al., 2017). In recent years, the role and importance of facilitating students’ voices have been highlighted, yet there is a clear lack of space for the emergence of such voices. In many instances, claims for acknowledging students’ voices remain occasional and tokenistic (de Leeuw et al., 2020; Young & Jerome, 2020). Dedicating space for the emergence of students’ voices not only reflects a commitment to inclusive education (de Leeuw et al., 2020) but, in the case of specific disciplines such as counselling, could potentially introduce other benefits such as those aligned with the adoption of a journey metaphor. By situating counselling students in spaces that facilitate transitional storytelling, further self-reflection opportunities emerge for them.

The COVID-19 pandemic had a big impact on students’ experiences across the globe. Students at different educational levels felt a sense of disruption, disengagement and disconnection from their educational contexts, resulting in the marginalisation of many students’ perspectives and voices (Hoskins & Donbavand, 2021; Wilson et al., 2020). With the virtualisation and hybridisation of higher education as a post-COVID-19 trend, the voices of students in certain disciplines could be in danger of further marginalisation, highlighting the need for practices that promote students’ voice, engagement and connectedness (Gourlay et al., 2021). Reduced opportunities for face-to-face engagement with peers, educators and supervisors could disrupt the provision of spaces through which counselling students previously voiced their journey experiences and concerns. Such spaces offered opportunities for formal and informal conversations that facilitate self-reflectivity and reflexivity, major requirements of quality counselling education, supervision and training (Dixon & Chiang, 2019; Granello & Young, 2019). Reduction of such spaces and opportunities and replacing them with individualised and detached learning spaces could reduce dynamic interactivity and reflexivity among students and potentially diminish opportunities that promote a sense of agency for some students. This is alarming for a discipline that aims to position itself among other emancipatory disciplines that highlight inclusive and ethical practice, social justice and sensitivity to issues of power (Lee, 2018). How do we promote a person-centred and reflexive approach in counselling practice, if we are not adopting a reflective and learner-centred practice in counselling education? Acknowledging counselling students as “contributors in the social construction of knowledge” and “using their [journey] experiences as relevant resources” (Silseth, 2018, p. 310) for improving counselling education curriculum and pedagogy could transform both the educational realm and its future generations’ practices. Students’ stories, voices and experiences are diverse. This highlights the need for attending to their local and particular knowledge, stories and subjectivities (Geertz, 1983). While voice-sensitive research processes make it possible to attend to such diversities, they could further promote reflective practices in counselling education and highlight counselling as a discipline that prioritises social justice (Lee, 2018). To honour local and particular knowledge and people’s subjectivities, narrative inquiry informed by theoretical underpinnings of constructivism and social constructionism informs this research.

### Narrative Inquiry: Constructivism and Social Constructionism

A narrative approach to inquiry embedded within a constructivist, social constructionist framework ontologically and epistemologically informs this research. Constructivism focuses more on internal cognitive processes by which every person makes meaning of experiences. Social constructionism considers the influence of wider contexts on how such cognitive processes shape. What goes on in the social context has varied influences on the shaping of different aspects of experience for each person (e.g. a social constructionist approach). Previous socialisations determine how a person reacts to new events, and how they story these developments (e.g. a constructivist approach). Learning and teaching research informed by such epistemological assumptions focuses on the facilitation of reflective possibilities for student participants to engage in storytelling. A narrative approach to inquiry prioritises students’ voice and provides opportunities for telling stories of evolving educational journeys. By telling stories, students deconstruct the social context, as well as the internal cognitive processes behind undertaking such journeys. They voice concerns and challenges against the backdrop of socialisations, initiations, passions and values that have been influential in shaping a decision to pursue a counselling career. Finally, by promoting a process through which students’ voices are storied, we extend an invitation for adaptation and social construction of new journey metaphors for counselling education. Achieving this is only possible by privileging students’ voices and collating stories of learning and teaching at a time when higher education itself is embarking on a journey. We acknowledge that by engaging in such research, we invite dialogue among counselling educators and researchers on many of the current higher education trends (e.g. virtualisation) that are insensitive to discipline-specific requirements. As such, this research aims to (1) explore and signify undergraduate counselling students’ voices in a rapidly changing and ‘virtualising’ educational world and (2) operate as a space of self-reflexivity for us as counsellor educators and qualitative and constructivist researchers who value reflective practice.

## Method

Being informed by constructivist and social constructionist epistemologies, this research qualitatively engages with counselling students’ transitional educational experiences. To do this, sensitivity to various voices that operate in students’ ways of sense-making about the transforming context of higher education and their ways of responding and adapting to such changes is essential. By adopting a qualitative approach through a multiple case study method (Yin, 2014), this research intends to explore and prioritise students’ voices and, by facilitating storytelling, get as close as possible to their ‘insider knowledge’ and ‘lived experiences’ related to their counselling education journey over the past 3 years (Yin, 2014). To respond effectively to the social marginalisation of a multitude of students’ voices, methodologies that capture a wide range of “experience-near” (Geertz, 1983, p. 57) narratives from different groups of people are required. Such research prioritises an ethics of care (Noddings, 1984) that carefully attends to participants’ subjectivities by facilitating voice-sensitive explorations. Voices present in students’ ways of storytelling about their journeys can be identified and highlighted (Brown & Gilligan, 1992). As such, the research operates as a voice-sensitive process in which counselling students story their transitions, intentions, aspirations, hopes, plans or values concerning studying a counselling degree, as well as their concerns, questions, challenges, confusions and, finally, feedback related to their educational journey.

### Sampling and Recruitment of Participants

After ethical approval was sought, purposive sampling was used to recruit participants. All final-year or recently graduated Bachelor of Counselling students in our program were informed about the possibility of participating in this research. Only four students volunteered to participate. As a nonprobability sampling technique, purposive sampling is justified due to the heterogeneity and scale of the population being studied (undergraduate students in Australia) (Merriam & Tisdell, 2016). It is important to consider that while the current research prioritised the task of attending to a multitude of students’ voices, it is not claiming an exhaustion of all possible voices that exist among our students and, as such, did not follow data saturation protocols. A small sample size of four participants (three females and one male) provided a better opportunity for the qualitative researcher to engage in a more detailed analysis of the participants’ stories and voices (Mauthner & Doucet, 1998). The Bachelor of Counselling program at Griffith University is represented by a majority of female students and 65% mature-age students who did not directly transition from school to university. The four participant students were all mature students with prior educational and work experiences in other fields. Each participant attended an interview that lasted between 60 to 85 minutes.

### Semi-Structured Interviews and Data Collection Procedures

The provision of a storytelling space which facilitates the emergence of a multitude of voices that operate within the participants’ various life contexts is aligned with the ethics of care (Noddings, 1984) that combats the marginalisation of voices. As such, narrative inquiry (Clandinin, 2013) was deemed appropriate and relevant, as it relies on researchers’ curiosities as the facilitator of storytelling (Clandinin, 2007, 2013). Semi-structured interviews are the most prominent form of collecting data for narrative inquiry (Clandinin, 2013), and they offer flexibility to facilitate subjective and rich storytelling so the researcher can learn about participants’ ways of sense-making (Clandinin, 2007). While these interviews provide a structure for the researcher to narrow the focus of inquiry, they also offer opportunities for the formation of story plots and themes. Through these interviews, participants could comfortably story aspects of their lived experience related to educational journeys and career plans. The narrative inquirer (interviewer—Peyman) was experienced with doing narrative inquiry, posed a curious and yet empathic manner of inquiry, positioning the participants as knowledgeable individuals with a wealth of stories and insights that matter (Clandinin, 2007).

The semi-structured interview intended to facilitate telling stories about the participant’s histories, hopes, values and aspirations behind wanting to study counselling, and their university and program experience such as concerns, challenges or complications that arise throughout the program as the university learning and teaching contexts transformed, up to the point of interview. Table 1 provides a summary of sample interview questions.

**Table 1:** A summary of sample interview questions

• When did you start the Bachelor of Counselling (BoC) program?
• What led to the decision of studying a BoC?
• What values and/or career ideas informed your decision to study a BoC?
• How did your significant people (e.g. parents, partner, siblings, friends) reacted to your decision of studying a BoC?
• What were some of the most surprising aspects of your studies when you first started studying a BoC (in your first year)? How did these evolve in the following trimesters?
• What were some of the best learning experiences of your studies so far?
• What were some of your most/least favourite courses in this program? What was it about them that was interesting and/or useful to you?
• How did COVID-related changes impacted you and your learning experience at first? What were the most challenging things you noticed?
• What were some of the most supportive factors/people (both in your personal life and within the university system) that facilitated your progress and made it easier for you to keep engaged in learning and developing your skills?
• As a result of being immersed in various courses throughout your program of study, what things might have changed for you? Both personally and professionally (e.g., as a future counsellor)
• To what degree, have your initial hopes, plans, expectations and values that motivated you for pursuing such a career in counselling have been fulfilled by your studies so far?

These questions reflect approximately one-third of the full interview protocol that was used. The interview protocol was developed through an iterative process of forming the first draft by the first author based on his reflections on teaching practices and previous conversations with the Bachelor of Counselling (BoC) students. Then, the second author reviewed the protocol and made some suggestions. Finally, another colleague with years of experience in higher education learning and teaching research reviewed the interview protocol and made further suggestions. Through this process, the interview protocol was modified substantially.

Each participant attended one interview with the first author. Only one of the interviews was face-to-face, and the other three were conducted online due to the COVID-19-related lockdowns. All interviews were audio-recorded and transcribed verbatim. Each participant was given a $25 gift card as a token of appreciation for taking the time to participate. This was justified as all four participants were mature-age students juggling work and family commitments (e.g. child and aged care) while studying. Participants were given the option to receive a fully de-identified interview transcript, to read and request changes or omissions, as well as a summary of the research findings prior to publication.

### Ethical Considerations

Participation in this research was completely voluntary, and students were informed that they could withdraw from the study at any point, without impacting their academic progress. While the interviewer was a previous lecturer for some of these participants, at the time of the interviews, he was not in an academic relationship (e.g. course convenor, program director, supervisor) with any of the participants. Also, since the participants were either graduated or in the final year of their studies, there was no possibility of any future academic involvement with the researcher in their current program. Given the small sample of participants, in addition to using pseudonyms, further details such as the participants’ age or gender may have been changed to protect their identity.

### Data Analysis

“Voice, as a polyphonic way of expression relates to the many different possibilities through which individuals [could] tell their stories” (Abkhezr, 2018, p. 129; Gilligan et al., 2003). In this research, the analysis of the interviews was intended to align with the research aims and the constructivist assumptions that underpin qualitative research. As such, a “voice centred relational method of analysis” (VCRA; Brown & Gilligan, 1992; Gilligan et al., 2003; Mauthner & Doucet, 1998) was chosen.

VCRA is a detailed and systematic analytical procedure that focuses on listening for participants’ multiplicity of stories and the polyphonic nature of their voice embedded in relational contexts. Through an exploration of voices present in the educational stories of counselling students, their “voices and perspectives [are kept] alive” (Mauthner & Doucet, 1998, p. 120). VCRA facilitates consideration of the wide range of relational, cultural and contextual factors that shape the construction of participants’ educational journeys in “an experience near manner while the interplay of various voices is considered” (Abkhezr et al., 2018, p. 21). The VCRA is different from traditional methods of qualitative data analysis that often intend to categorise, themify or quantify the interview text (Tolman, 2001). Through VCRA, the researcher intends to prioritise the voices of a particular individual in a particular context by listening to them from different perspectives. As such, the VCRA is a “Listening Guide” (Gilligan et al., 2003) and does not follow a data saturation protocol. This analytical approach is consistent with the “ethics of care” (Clandinin, 2007, p. 30; in Noddings, 1984) that highlights attending to participants’ subjectivities and voices without a reductionist agenda. VCRA consists of four stages of engaging with qualitative data. To adopt the required analytical lens for each stage, multiple transcript readings and audio-recording listenings occurred. Every stage was completed independently, and a comprehensive report of the four stages was developed for each participant.

### Role of the Researcher

Narrative research emphasises the relational nature of research and highlights the importance of a reflexive researcher. This includes acknowledgment of the researcher’s social location, background and even emotional responses to the respondents’ stories (Brown & Gilligan, 1992). Reflecting on such acknowledgements also aligned with the second aim of this research, which was the facilitation of a space of self-reflexivity for us as counsellor educators and qualitative researchers. To achieve this, the lead author who conceptualised the research project recruited the participants, conducted the interviews, completed the bulk of the analysis and engaged in various reflective procedures at different stages of data collection and analysis. As a non-white, middle-aged, migrant, male counsellor educator, I (Peyman) acknowledged many of my shared commonalities and yet differences with the participants. Reminding myself about my particular journey in the counselling field, I had to constantly reflect on my biases when hearing stories that resonated (or not) with some of my own perspectives, values or interests (e.g. being more interested in certain stories). Early realisation of the pervasive influence of my potential biases before I delve into data collection attracted me to consider voice-sensitive research and the VCRA for data analysis. I believed and hoped that such a process through which the participants are actively invited to tell stories of learning and teaching experiences in the backdrop of their initial hopes and commitments to pursue a counselling career could produce a dialogic space which was also emancipatory (e.g. giving them a stage). I hoped this practice could be replicated in counselling education.

### Trustworthiness

Trustworthiness ensures rigour and consistency in qualitative research. Many strategies were employed to substantiate research trustworthiness. Generally, criteria suggested by Lincoln and Guba (1985) were used to enhance trustworthiness. From the conception of research topic to data analysis, the research team met regularly to discuss and reach consensus on recruitment, the interview protocol and the analytical strategies. Other strategies included offering voluntary member-checking to the participants. Only one participant requested to read the de-identified transcripts and, upon checking, did not request any changes. Despite this, all four participants received a summary of the research findings prior to publication. Furthermore, the lead researcher used several reflexive processes prior to and throughout the different stages of research conception, data collection and data analysis to enhance confirmability. Some of these reflexive processes and their outcomes were explained in previous sections (e.g. role of the researcher and the interview protocol).

## Findings

Participants’ stories consisted of a range of values and plans that initially informed their decision to transition into a BoC. These stories also reflected how such values and plans survived, transformed or operated later in managing through various educational transformations and complexities, including challenges related to the COVID-19 pandemic. Participants’ stories occurred in diverse contexts and revealed the role of numerous relational and contextual influences in their journeys. First, a brief introduction about each participant (pseudonyms are used) and their context is provided. Then, to reflect on the participants’ stories, while keeping their voices alive, the findings section of this paper is divided into four sub-sections, each dedicated to one of the four VCRA stages of data analysis, namely the plot, the I-poems, the relational context and, finally, placing participants within their contexts and social structures. The analytical procedures, strategies and considerations that were unique to each stage of data analysis are described under its stage, followed by each stage of analysis findings from the participants.

### Participants’ Background Stories

#### Rebecca

Rebecca, a single mother with two children in her “mid-to-late forties”, began her studies in 2019. At the time of the interview, Rebecca was starting her third and final year of studies, while doing placement. She worked as a photographer for years (self-taught). Part of her reasons to study counselling was related to her priorities of spending more time with her daughters. Her initial ideas included a graphic design and/or social media career, but certain incidents and reflections changed that. She completed a Counselling Diploma prior to attending university. She is already seeing clients in her private practice but is considering working part-time for a community organisation after graduation.

#### Claire

Claire, in her early forties, began her studies in 2019 and was doing her first placement at the time of the interview. She has two previous tertiary qualifications, including a Bachelor and a Graduate Certificate of Applied Science. She was a laboratory scientist for years which did not give her much room for social interactions. This partially informed her career transition to counselling. This transition was not supported by all the important people in her life and resulted in some relational tensions. While still working part-time in her previous field, she is actively volunteering to prepare for work in the counselling field.

#### Mike

Mike, in his late forties, was the only male participant (with English as second language). With a Diploma in Management, Mike partially did a few other degrees without completing them. Years ago, he moved from Brazil to Australia while working in a managerial position for more than 10 years in both countries (same company). Not being satisfied with the level of human connection in his work, Mike decided to make a transition and pursue a different career in a field that aligns more with his humanistic and social values. In 2018, with his daughter, he began studying a Bachelor of Psychology (Hons), but later decided to switch to the BoC. Mike graduated from the BoC a month before the interview.

#### Shana

Shana, in her early sixties, started her studies in 2018 and graduated a month before the interview. With a Diploma in Beauty Therapy, Shana worked for 20 years in that area. Over time, realising some of her skills in listening to and supporting people, she pursued a Diploma in Counselling while working as a telephone counsellor (suicide prevention) for over 12 years before university. Supported by her husband and children, she considered her long-standing passion for teaching as an influential factor for her decision to study counselling. Being the first in her family to graduate from university, and valuing family life and supporting other families, Shana predominantly prefers to work with children or other areas such as domestic violence.

### Stage 1: Listening for the Plot

To reveal plot/s, the first stage of VCRA analysis focuses on “what is happening or what stories are being told” (Gilligan et al., 2003, p. 160) by the participant. As such, major events, actors and the social context in which the plot occurs are noted. Therefore, each participant’s broader stories were considered to thicken and form an overarching plot (or plots) in relation to the decision of studying a BoC, and later being faced with an unexpected hybridisation process. By listening to the participants’ story plots, similarities and differences in terms of how each one narrated a journey were explicated.

#### Rebecca

In listening to Rebecca’s stories of deciding to study at university after working as a freelance photographer, a sense of developing competence and self-confidence emerged. The plot was about eventual self-realisation despite the odds. The genre was of overcoming and dominating doubts (mostly seeded by others around her) which were questioning the initiation of a new journey. In telling stories of motivations for studying counselling, there were initial sub-plots of being forced to measure up to what was referred to as a “pedestal” (university studies) and comparing herself negatively with other mature-age students’ journeys and their successful transitions. However, the plot reflected the eventual shift, as stories of transitions within university during COVID times were filled with a sense of self-worth; recognition of abilities, such as organisation and communication skills; and even a sense of pride for studying alongside psychology students.

#### Claire

An emotionally tense plot was present in Claire’s stories of transition into university. Following the big decision in her life to transition from a science lab to study counselling, her parents disagreed with her choices and devalued her bravery. A “burden” overcast her university performance. It felt as if Claire had to constantly fight for her decision, a fight to ensure the visibility of her success. Out of this fight emerged a theme of situating self into a career path that accommodates desires for “increased human connections and social interactivity”. However, like Rebecca, doing well in her courses and making friends at university disrupted the emotionally tense plot. Voices that echoed possibilities for connections and progress at university gained more stage. Claire talked about her satisfaction with the university context providing opportunities for social interactivity prior to pandemic. Reaching out, meeting up and forming groups with other BoC students were functions that validated her decision of wanting to break out of the isolating lab. However, Claire identified the COVID-related learning and teaching changes as “so jarring”. The hybridisation tremendously disrupted Claire’s learning expectations, as they reproduced a similar isolating work context from which she was hoping to move away: “… a screen between [me and] that passion, I can’t feed of any excitement for the subject material anymore … can’t ask any immediate questions – there is a two-day process to get a response that removes the immediacy of learning”.

#### Mike

Unlike the previous two participants, Mike’s stories of career transition to counselling had a theme of support and encouragement. Working for more than 10 years in a company that his father also worked in for a lifetime appeared to be not aligned with Mike’s values anymore. Emphasising that “working with people has always been [his] strength”, Mike highlighted the value of his transition decision. Stories of commencing university studies and immersing with others were filled with themes of motivation, joy and realisation of long-standing passions. Themes of freedom and choice later emerged at university. Mike stated that such new learning opportunities and connecting with others at university “allowed me to realise that I can do other things … that I am free … I can choose …”. In telling stories of his learning journey at university, Mike had a more direct approach to outlining positive and negative experiences of his BoC experience. He was the only participant who provided some actual program expectations and feedback and was critical of certain aspects of the program, such as the heavy psychological focus and lack of social and cultural content. Mike had a sense of ‘voicelessness’ and “what can I do about it” on COVID-related changes that affected learning and teaching. A plot of ‘being tested for his adaptation skills’ and ‘the need to remain resilient’ in the face of these changes emerged. He considered these the second wave of adaptation efforts, after the initial adaptations that he made upon joining university. However, he stated: “nothing compares to that face-to-face delivery … it always opened a space for dialogue … [the online space] doesn’t give as much a chance for people to engage”.

#### Shana

Shana’s plot was one of appreciation, surprise, fulfilment and loss. Anticipating to study counselling at university for a long time, Shana finally believed she could rely on skills she used in her previous career which are essential for a counsellor. She was encouraged by her family to make this transition, and later when she needed to adapt to COVID-imposed circumstances. Shana was appreciative of her family, as well as the “impactful support” provided by their program director throughout her studies. Shana’s stories of her first days at university had a theme of surprise as she met many other mature-age students and was feeling more relaxed about not being the only one. The grandness of the university context and the number of psychology-related courses in the BoC program substantiated the surprise theme. At many points in her interview, Shana emphasised the importance of making “lifetime friends” as a positive outcome of studying a BoC at university. Shana’s sense of fulfilment was also related to her views on the experience of BoC broadening her perspective on matters of cultural diversity and aboriginal perspectives in counselling. Finally, on COVID-related learning and teaching changes, Shana highlighted “loss of connection”, the “disappearance of anticipation” and “being at a loss” with the university friendships that could enhance her learning journey.

### Stage 2: Listening for ‘I-poems’

Since the relational method emphasises distancing the researcher from objectifying participants’ voices, this second stage of analysis focuses on the voices of “I” and tuning into the I-positions of each participant in describing self in context (Gilligan et al., 2003, p. 162). This listening aims to get us closer to the participants’ “multi-layered” (Mauthner & Doucet, 1998, p. 130) and “contrapuntal” (Gilligan et al., 2003, p. 164) voices that occur simultaneously. The concept of contrapuntal voices refers to the polyphonic (and not monotonic) nature of the human psyche, like the multiple voices in a musical concert. Every single voice co-occurs with other voices and has a particular relationship (e.g. tension, support, opposition) with other voices of self, others or heard or shaped within a particular cultural or contextual social structure (Gilligan et al., 2003).

Development of “I-poems” involves collecting all pronouns of “I” along with their following descriptive phrase and placing them sequentially into a different document to form what shapes a series of I-statements (aka I-positions). These I-poems assist the researcher to highlight participants’ subjectivities by tuning into instances when shifts occur in their narratives (Brown & Gilligan, 1992). This makes it possible to distinguish and listen for “cadences and rhythms” and the different voices that shape each participant’s expressions of self in stories (Gilligan et al., 2003, p. 162). By tuning into the I-poems of the participants, different voices emerged in each participant’s stories. Some examples are reported in this section for three of the participants as reporting the entire list is beyond the limits of this paper. Table 2 provides brief samples from three of the participants’ I-positions that emerged from segments of their transcripts.

**Table 2:** Emerging voices from participants’ I-poems

Participants’ voices	I-poems
Rebecca’s voices of ‘dependency and reliance’ vs. ‘overcoming doubt’	*I didn’t think / I had a breakup / I didn’t think I would / I was a single mum / I was relying / I just got to thinking / I really wanted to / I started looking / I wanted to do / I was photographing / I could be / I’m sort of stuck going down / I got a cold call / I was sitting / I went Oh no / I saw a lot of study / I started thinking / I really wanted to be there / I wondered how I would do that / I thought about counselling / I could dip my / I got into / I went like ok / I wanna be that person / I want to do that / I got to thinking / I was really good at listening / I kind of got a buzz / I could sit there / I know that / I find that / I feel like I’ve been there / I’ve done / I was like “Ok, I can” / I could / I wanted to / I could build up / I was noticing / I’m thinking*
Claire’s voices of ‘desiring connection’ vs. ‘treachery’	*I did some reflecting / I found that transition really hard / I’ve done a bit of reflecting / I was like why / I finding this hard / I find it so jarring / I started out / I picked counselling / I was a scientist / I was talking to plants / I was talking to worms / I was dissuaded from talking / I felt very isolated / I self-sabotaged myself / I realised / I really wanted to do / I really want to connect / I picked counselling / I am here / I’m going through / I love going to lectures / I love connecting / I’ve been thrown back*
Mike’s voices of ‘disenchantment and responsibility’ vs. ‘freedom and possibility’	*I think / I had the biggest struggle / I started university / I went to those employment / I saw that consultant / I tried everything / I could / I had a big struggle / I was coming in / I had a bit of trouble / I have always been fine / I have always been able / I need it / I’m OK / I would say / helped me get through / I say / I used to work / I would work / I decided to leave / supported me from day one / make you happy / I don’t know how / I’m 48 / I’m responsible / do you need any / you pay your bills / You know / be there for me / if I was still a young man / starting my life / that moves me / find myself again / I can do things / I am free / I can choose / I can make my destiny / I will be able / I am passionate / I can hopefully*

#### Rebecca’s Voices of ‘Dependency and Reliance’ vs. ‘Overcoming Doubt’

As Rebecca storied how her decision of joining university was not a smooth transition, a few voices interacted with each other at different levels. Some I-poems portrayed Rebecca as the ‘single mother after a relationship breakup’ who was trying to retreat and find her sense of self. Such voices soon gave space to a sense of determination for ‘overcoming doubts’ and anticipating a future of possibilities. In this transformation, Rebecca’s voices of *dependency* and *reliance* transformed to voices of *overcoming doubts*.

#### Claire’s Voices of ‘Desiring Connection’ vs. ‘Treachery’

For Claire, different voices were operating when the online learning transitions occurred. The transition became further challenging as it re-introduced an isolating context from which Claire intended to distance herself by initiating university studies in a relational field such as counselling. Claire reflected that her sense of trust in the career path that will fulfil her desire for human connection might have been betrayed. As such, the voices of *treachery* were in disagreement with voices of *desiring connection*.

#### Mike’s Voices of ‘Disenchantment and Responsibility’ vs. ‘Freedom and Possibility’

For Mike, who started university after more than 10 years in management, the university transition brought him to a crossroad with finances, finding new employment, and unexpected support from his father, who worked for 40 years in the same company and always wanted the same stability for his son. Now after leaving the company to pursue studies, Mike felt he might have let his father down. However, while struggling for nearly a year to find employment during his studies at university, Mike was surprised and moved by his retired father’s encouragement and offer of financial support. Voices of *disenchantment* and *responsibility* were situated at a crossroad with voices of *freedom* and *possibility*.

### Stage 3: Listening for Relationships

Stories make sense only in a relational context. As we engage in storytelling, we make sense of our experience by relating to others. This stage of the VCRA focuses on exploring participants’ experiences of self in the “relational landscape of life” (Brown & Gilligan, 1992, p. 16). The researchers tune into the participants’ “ways of relating to other people” (Abkhezr et al., 2018, p. 21). All words and sentences that indicate relating and interacting with other people were highlighted in transcripts, constituting a relational list for each participant. The intention was to “explicate the nature and dynamics of relationships” and to “expose the relational dimensions of each participant’s stories” (Abkhezr et al., 2018, p. 21). Participants storied a range of relational experiences that informed their decision to enter university as mature-age students. Throughout their time at university, such relational stories were further extended as students navigated a new social context. Table 3 provides a summary of some of the relational territories that were highlighted by the participants.

**Table 3:** Major relational dimensions and themes

Participants	Relational dimension and themes
Rebecca	Family context both supportive and yet tense about career transition Other similar-age females validating transition decision and overcome uncertainty Future generation of counsellors and younger BoC students Peer support helpful for self-reflection and clarification of counselling journey, of university’s broader relational context as a stage for the development of new perspectives and worldviews Making close friends at university COVID and online learning severely disrupting relationships, learning and valued socialisations
Claire	Career transition primarily initiated due to the importance of relational work Forming communities of figuring things out with other BoC students Husband supportive of career transition but parents strongly against University relationships help for reflecting on self and values/intentions
Mike	Career transition primarily initiated due to importance of relational work Co-travelling with daughter in university transitions Family support for transition into university Overseas father’s support motivating in a complex way Forming communities of practice with counselling students Relating to others with sensitivity to diversity and acknowledgment of different worldviews COVID and online learning severely disrupting relationships, learning and valued socialisations Concern for others who study counselling yet they need help in their personal life Views on relating to Bachelor of Psychology students
Shana	Relational context of the family since childhood and a culture of supporting family members Supporting her children with their life and care for grandchildren Needing to work and not retire to be able to support family Relational commitments fuelling transition decision Family support for transitioning into university and later for COVID adjustments Surprised by peer support and finding other mature-age friends at university Relating in various ways with teaching staff

### Stage 4: Listening for Placing Participants Within Cultural Contexts and Social Structures

VCRA’s final stage aims to find a place for the participants’ “accounts and experiences within broader social, political, cultural and structural contexts” (Mauthner & Doucet, 1998, p. 20). This stage aims to reveal the role of dominant macro-level narratives, power structures and influential cultural norms and societal values in each participant’s journey. This final VCRA stage provides a scaffolding for all the previous stages, to establish a ‘placeholder’ for the many different voices and relationships that have emerged so far. Major areas that emerged in participants’ stories that provided a performative stage for the operations and interactions of all voices and relationships included the following: (1) the immediate family members’ (including partners, children and parents) reactions to a mature-age student’s career change and transitioning into university; (2) extended family members’, relatives’ or social networks’ ways of responding to such transitions; (3) support or lack of support from the previous two groups while studying at university which could include financial or social support; (4) the university context and the two relational domains of interaction with peers (that includes peers who began earlier as well as those at the same level of entry) and academics and university staff; (5) the dominant narratives reflecting psychology programs as more legitimate and worthy, hence on a “pedestal”; and (6) the COVID-19 pandemic, its unexpected changes and a consequential marginalising impact.

### Cross-Participants’ Contextual Findings

Finally, to conclude the findings, some detailed comparisons across the four participants are reported. While reiterating that the intention is not to reproduce generalisable grand narratives about counselling students in different contexts and with different needs, this section outlines some potential synergies, similarities or differences that existed among the four students and to follow the common practices in narrative research where provision of rich and thick descriptions about the participants’ lives are valued (Clandinin, 2013). In listening to Claire and Rebecca, the role of significant people in their lives (e.g. parents, partners, siblings) who were not agreeing with their career transition decision seeded doubts and was reproductive of dominant societal stories, questioning self-worth, abilities and capabilities if one is not fulfilled with their established career. Such relational spaces led to emergence of a different context, operating in opposing ways for Mike and Shana who were supported by their significant others.

In telling their stories of transition to university and deciding on a program to study, three of the participants (Rebecca, Claire and Mike) mentioned that they would have studied a Bachelor of Psychology if the timeframe for registration as a psychologist in Australia was not so long. This, along with other comments across the interviews, was reflective of the operations of societal power structures that legitimise certain disciplines as more fit for ‘mental health practice’. However, participants also highlighted that after a few trimesters in the BoC program, they were pleased with their decision as they perceived the counselling field as more practical, targeted on skills development, and hands-on.

Finally, although one major aspect of inquiry was related to students’ feedback on the program and their experiences of it as a whole, not many suggestions or reflections were made by the three female participants. Mike was the only participant who raised some issues. He was disappointed by the quality of peer feedback they received throughout the program and wished for more direct and even harsh feedback when needed. He also expected the inclusion of more sociological, cultural and spiritual concepts and considerations that could enrich counselling education and practice, instead of a heavy psychological focus. Mike shared that he enjoyed the learning that emerged for him as a result of engaging with reflective assessment items throughout the program. Shana also highlighted how she believed that the BoC was helpful for her to learn more about diversity, cultural issues and sensitivities required in working with aboriginal communities. Shana highlighted that this was achieved for her as she learnt from an Aboriginal and Torres Strait Islander peer in the program, subsequently leading to a placement with a centre that supports Aboriginal and Torres Strait Islander youth. These final notes make it easier to develop a richer integration between the multilayered accounts of each participant’s story (the plot, the voices and the relationships) and the contexts through which these stories are told and lived.

## Discussion

Against the background of the COVID-19 pandemic and its impact on the higher education sector, it was timely to explore Bachelor of Counselling students’ transitional stories of studying a program that aligns with their values and stories of survival and development. At a time when students’ voices are in danger of marginalisation and voice-sensitive research is encouraged to promote student engagement and connectedness (Gourlay et al., 2021; Hoskins & Donbavand, 2021; Wilson et al., 2020), this research attempted to situate the voices of these students within the higher education’s transforming context as it re-emerges through a turning point of survival and adaptation. The interviews informed by narrative inquiry provided an opportunity for these participants to narrate stories of navigating the counselling program through which their voices were carefully attended and then analysed by VCRA, reflecting the commitment of this research in adopting an ethics of care (Noddings, 1984) for marginalised voices and subjectivities.

For the first time since the participants began their counselling studies, they had a chance to voice their stories of transitioning into university with the aim of becoming counsellors and, yet by chance, navigating the higher education context at a remarkably fluctuating time. For this purpose, interviews based on the foundations of narrative inquiry facilitated storytelling. VCRA prioritised the subjectivities of these students and their voices, which were embedded in a relational and contextual world. By storying their transitions into university and a mature-age student survival in a rapidly shifting higher education context in which counselling education faces complexities as a result of the pandemic, these students highlighted the importance of socialisations, social structures, dominant narratives and relationships for their developmental journeys as counsellors (Aristovnik et al., 2020; Li et al., 2021; Potra et al., 2021; Toquero, 2020).

The findings highlighted stories of values, career ideas and hopes, personal and professional developments and ambitions, learning and teaching, and resilience and survival that were particularly important for the discipline of counselling, counselling education and training, which could also be useful for other skills-based or relational disciplines. Overall, these findings informed by the VCRA revealed aspects of the participants’ journeys in three broad categories, including (1) the counselling discipline as a relational career development choice, (2) supportive and disruptive voices and relational structures in both personal and university contexts and (3) COVID-19-induced hybridising process disrupting students’ learning and teaching experiences and expectations. Each will be discussed briefly.

### The Counselling Discipline as a Relational Career Development Choice

Counselling practice is built upon its interpersonal and relational dimensions. Such relational focus has been historically emphasised as the major bedrock of counselling efficacy and outcome (Duff & Bedi, 2010; Wampold & Imel, 2015). This same relational characteristic informs the career plans of many who enter such a field. The decision to study counselling at a later stage of life, in the form of a career transition, can be viewed as a relational career development choice. Such a choice is informed by long-term and sometimes lifetime acquired values, through reflecting on previous life and work experiences.

### Supportive and Disruptive Voices and the Relational Structures

The findings highlighted the role of different supportive and disruptive relational structures and voices that influence mature students’ situatedness in a wider and more complex web of social, relational and cultural contexts (e.g. previous/current work and family environments, dual caring responsibilities for children and parents). Transitioning into university to study a BoC and to initiate a career change at a later life stage following the pervasive influence of such supportive or disruptive relational structures could indicate a certain level of expectations from this educational journey to result in a sustainable career with enhanced employment opportunities, for which a relational educational space is vital.

Deconstructing the operation of various voices and relational structures that inform, challenge, disrupt or support students’ initial decision to choose and transition through a BoC program revealed the role of various dominant narratives and power structures. For example, the position of the BoC program among other programs was highlighted by students, as they storied experiences that reflected on the existence of a hierarchical power structure among the programs. These stories potentially reflect other societal discourses and contextual factors such as more income, recognition or legitimacy and power assigned to psychologists compared to counsellors in Australia.

### COVID-19-Induced Hybridising Process Disrupting Students’ Learning and Teaching Experiences and Expectations

The COVID-19 pandemic disrupted students’ experiences of higher education in many ways (Aristovnik et al., 2020; Hoskins & Donbavand, 2021; Wilson et al., 2020). The teaching of particular practical disciplines, such as counselling, faced significant issues associated with online teaching and learning (TEQSA, 2020). Despite certain opportunities for counselling practice, education and training, unique challenges were encountered by both educators and students (Tudor et al., 2021). Participants storied that the digitising and hybridising processes that disrupted their learning experiences created relational distances which were contrary to many of their expectations from such a program. Participants reflected on the value of traditional activities that were replaced by online and digital teaching practices (e.g. large lectures, small tutorial groups, online role-play assessments, consultations with the teaching team, peer group activities) and the online arrangements’ limited opportunities to foster reflexive skill-development journeys (Dixon & Chiang, 2019).

### Implications

This research has important implications not only for counselling education and training, and the specific program and its students, but also for the broader context of higher education in Australia. It also impacts the researchers as educators situated in a context in which multiple forces inform and shape their practices. Furthermore, potential implications for students are also outlined.

#### Counselling Education and Training

Through voice-sensitive research that facilitated students’ storying of their learning and transition journeys, this research acknowledged students’ contributions to the social construction of knowledge that emerges from their journey experiences as relevant resources for improving counselling education curriculum and pedagogy (Silseth, 2018). Reflecting on students’ learning experiences and journeys (Inkson, 2015) by attending to their voices has serious implications for counselling educators and supervisors who are engaged in designing and delivering counselling programs (Taylor, 2020). We extend an invitation for the adaptation of new journey metaphors for counselling education that are aligned with its relational foundations and social justice roots (Lee, 2018). One major practical implication for counselling education is related to students’ feedback on the course and program development. Ensuring that student voices are part of an ongoing feedback process between students, programs, institutions and accreditation bodies will allow for greater emphasis on meeting the training standards, including reflective opportunities to support student development. Such consideration of students’ feedback in their rapidly evolving learning contexts could also inform counselling training accreditation requirements, and the quality assurance and enhancement processes more generally for higher education in Australia. However, prioritising students’ voices, and acknowledging the relational nature of their counselling journeys, necessitates the provision of quality relational and reflective feedback opportunities. This could involve moving beyond standard written feedback and providing opportunities for consistent feedback roundtables, peer group feedback meetings or one-on-one feedback interviews about the program. More specifically, further context-sensitive research like this could enrich the development and improvements related to counselling programs across the country or even internationally. To enrich and extend practice-based evidence for counselling education and training, one strategy could be the introduction of a requirement for all counselling programs to provide regular student feedback, prepared by a group of students to the accrediting bodies (PACFA, 2022).

Another practical implication for counselling education relates to the prioritisation of pedagogies that value relational practices in counselling education and training. Meeting students’ expectations regarding such a relational career choice means that curriculum designers and teaching staff need to prioritise pedagogies that value relational practices in counselling education, such as facilitating ongoing interactions between peers and the supervisory-teaching teams about the students’ journeys and the relational aspects of such journeys. This can emphasise the importance of client-counsellor relationship by valuing the interpersonal dimensions of counsellor-supervisor-educator relationships (Wampold & Imel, 2015). Finally, since the counselling discipline, compared to many other disciplines, often attracts more mature-age students, students’ choice of studying in a relational field and the interaction between such a choice under the influence of dominant power and relational structures need further attention (Faris & van Ooijen, 2011; Tomm et al., 2014). Therefore, educational spaces that foster a greater degree of continuous self-reflectivity and reflexivity are deemed more appropriate (Dixon & Chiang, 2019) to help these students constantly review their evolving stories, career development goals and expectations, in the face of pervasive power structures, so that their evolving needs are clearly communicated with the education providers and supervisors.

#### The Specific Bachelor of Counselling Program

We reflected on possibilities and considered strategies to minimise the adverse impacts of a rapidly hybridising educational context on our counselling program. We actively pursued new initiatives to provide opportunities for students to engage with peers and the teaching team in face-to-face environments. Following our research, two new courses specific to the BoC program were developed and offered to increase students’ engagement among other goals. After reviewing the needs of our students and the accreditation requirements, we decided to make both courses fully on-campus and face-to-face. Overall, student feedback on the on-campus and face-to-face nature of the two courses indicated a high level of satisfaction.

#### The Individual Researcher

The findings of this study also have implications for the two researchers in terms of their ongoing research and learning and teaching practices. First, the researchers have been informed through enhanced reflexivity by engaging with first-hand, experience-near stories of participant students about their journey of becoming a counsellor. This could serve to inform and modify the researchers’ future teaching practices to be more responsive to students’ needs, and to inform the ongoing program quality enhancement processes that are shared with colleagues. With a better grasp of the limitations or challenges that the students face throughout their studies, the researchers could also advocate for the needs of future students or adapt the program, as stated through the above example of two new courses, where it is possible to meet those needs. Second, the study has implications for the researchers in terms of contributing to a scholarship of constructivist learning and teaching. For example, the researchers engage in reflections on what aspects of the research design were helpful in meeting the research goals (e.g. providing a space for voices to emerge), and what could improve, or were not aligned with the aims or the epistemological underpinnings that the research intended to adopt. This will inform the researchers’ future research initiatives.

#### The Students

Finally, since students had the chance to story their career and educational journey, the overall research participation experience operated as a voice finding process through which they may have developed further agency in relation to their studies, training and future practices (Abkhezr et al., 2020). As such, by facilitating storytelling on learning and teaching experiences and the journeys of becoming a counsellor, students were provided with a space in which some of their potentially marginalised and forgotten voices emerged. Such emergence of voice was reflective and, in some cases, revealing of possibilities for actions that were informed by the participants’ realisation of values, hopes and skills.

### Limitations and Considerations

One limitation was related to a small sample size and only one interview with each participant. Spending more time with the participants could contribute to the emergence of further voices. The differences between participants who graduated and those who were still studying and how the researchers’ dual roles (as both convenors and researchers in this context) could limit freedom of expression for participants and disrupt credibility must be considered. Future research could consider a larger group of participants (e.g. different age groups or at different stages of their educational journeys), more interviews with each participant and providing participants with time for reflection between the interviews, and also cross-program comparisons with psychology students, or graduate and undergraduate counselling students.
